# The Antioxidant Activity and Their Major Antioxidant Compounds from *Acanthopanax senticosus* and *A. koreanum*

**DOI:** 10.3390/molecules200713281

**Published:** 2015-07-22

**Authors:** Young-Hyun Kim, Myoung Lae Cho, Dan-Bi Kim, Gi-Hae Shin, Jin-Ha Lee, Jong Seok Lee, Sun-Ok Park, Sang-Jong Lee, Hyun Mu Shin, Ok-Hwan Lee

**Affiliations:** 1 Department of Food Science and Biotechnology, Kangwon National University, Chuncheon 200-701, Korea; E-Mails: vvkyh@naver.com (Y.-H.K.); meanglae@naver.com (M.L.C.); danbekim22@nate.com (D.-B.K.); cordelia162@hanmail.net (G.-H.S.); tre98@hanmail.net (J.-H.L.); 2 National Institute of Biological Resources, Incheon 404-708, Korea; E-Mail: jongseoklee78@gmail.com; 3 STR Biotech Co., LTD, Gangwon 200-160, Korea; E-Mails: lovastar@strbiotech.co.kr (S.-O.P.); lsj@strbiotech.co.kr (S.-J.L.); 4 Department of Pathology, University of Massachusetts Medical School, Worcester, MA 01605, USA; E-Mail: hyunmu.shin@umassmed.edu

**Keywords:** *Acanthopanax senticosus*, method validation, antioxidant activity, chlorogenic acid, caffeic acid

## Abstract

The antioxidant activity and chlorogenic acid and caffeic acid contents were investigated from different parts of *Acanthopanax senticosus* and *A. koreanum*. Antioxidant activity was assessed by various *in vitro* assays such as DPPH, ABTS, FRAP, reducing power assays and ORAC, and the chlorogenic acid and caffeic acid were validated by HPLC chromatography. Among the various extracts, the fruit extracts of *A. senticosus* and *A. koreanum* exhibited strongest antioxidant activities including ABTS, FRAP, reducing power and ORAC, however, strongest DPPH radical scavenging activity was observed from the leaf extract of *A. senticosus*. In addition, the antioxidant activities of various extracts were correlated with total phenolic and proanthocyanidin contents. The major phenolic contents from various parts of these plants observed that leaf extract of *A. senticosus* expressed higher levels of chlorogenic acid (14.86 mg/dry weigh g) and caffeic acid (3.09 mg/dry weigh g) than other parts. Therefore, these results suggest that the leaf of *A. senticosus* may be an excellent natural source for functional foods and pharmaceutical agents, and the validated method was useful for the quality control of *A. senticosus*.

## 1. Introduction

The *Acanthopanax* species, belonging to the Araliaceae family, are widely distributed in the Far Eastern region of Russia and North-East of Asia countries such as Korea, Japan, China, and known as powerful tonic and medicinal herbs. These herbal plants have been attracting attention in the last few years because of their strong bioactivities, harmlessness, and low side effects [1,2,3,4,5]. *Acanthopanax senticosus* is called *Eleutherococcus senticosus*, Siberian ginseng, or Gasiogapi in Korea. A lot of studies reported that *A. senticosus* and *A. koreanum* contained various bioactive constituents, including triterpenoid saponins, lignans, coumarins, flavones and phenolic compounds [6,7]. The elutheroside B and E are major active lignans of *A. senticosus* and *A. koreanum*, which have immunomodulatory, antioxidant and anti-inflammatory activities [6]. In addition, other phenolic compounds contained these herbal plants, such as caffeic acid and chlorogenic acid also showed strong antioxidant ability *in vitro* and *in vivo* assays [8,9]. Recently, our previous study found that the stem of *A. senticosus* and *A. koreanum* contained higher amounts of lignans (eleutheroside B and E) than other parts of their plants [10]. The extracts of root, fruit, and stem are commonly used as ingredients in healthcare and medicinal products. Moreover, other parts of plants, such as the leaf, also could be useful in the food and pharmaceutical industries because of the high amount of their constituents.

To evaluate the quality of herbal medicines, reliable analytical methods have to be applied to the quantitative determination of the constituents with known therapeutic activity or to the final products [11]. Phenolic compounds, especially chlorogenic acid and caffeic acid, are major active compounds from *A. senticosus* and *A. koreanum*, however, few studies have determined the chlorogenic acid and caffeic acid content of *A. senticosus* [12,13]. Therefore, there is need to quantify it by validated methods in these herbal plants. The purpose of this study was to investigate antioxidant activities and correlation between antioxidant activities and total phenolic, flavonoid, and proanthocyanidin contents from different parts of *A. senticosus* and *A. koreanum*, and to determined chlorogenic acid and caffeic acid contents in the various parts of these plants. In addition, the chlorogenic acid and caffeic acid from the leaf of *A. senticosus* were validated using high performance liquid chromatography (HPLC).

## 2. Results and Discussion

### 2.1. Antioxidant Activity from Various Parts of A. Senticosus and A. Koreanum Extracts

**Figure 1:**
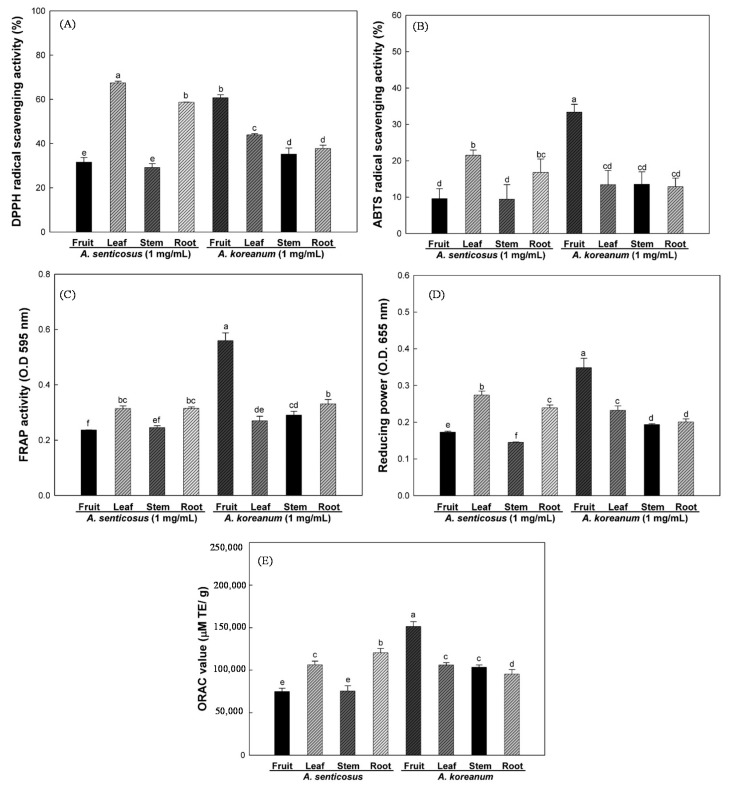
DPPH radical scavenging activity (**A**); ABTS radical scavenging activity (**B**); FRAP activity (**C**); Reducing power (**D**); and ORAC (**E**) in the different parts of *A. senticosus* and *A. koreanum*. Each bar represents the mean ± SD of triplicate determinations, *n* = 3. Statistical analysis was performed using one-way ANOVA (*p <* 0.05).

Reactive oxygen species (ROS), defined as the imbalance between ROS and antioxidants in favor of the oxidants, has been suggested to be the cause of aging and various diseases in humans. Therefore, antioxidant therapy is vital in scavenging free radicals [14]. The antioxidant potential of various extracts of *A. senticosus* and *A. koreanum* was determined by the 2,2-diphenyl-1-picrylhydrazyl (DPPH), [2,2ʹ-azino-bis (3-ethylbenzothiazoline-6-sulfonic acid)] (ABTS), ferric reducing antioxidant power (FRAP), reducing power and oxygen radial absorbance capacity (ORAC) assays. As shown in Figure 1, the fruit extracts of *A. koreanum* have markedly higher ABTS, FRAP, reducing power and ORAC activities than those of other parts of *A. senticosusand* and *A. koreanum* extracts at the concentration of 1 mg/mL. However, DPPH radical scavenging activity of various extracts exhibited that the leaf extracts of *A. senticosus* are much greater than other part extracts. The mechanisms of antioxidant action in radical scavenging assay differed from those in the FRAP, reducing, and ORAC assays [15]. Similar results have reported that the three tropical fruits and wild herb extracts also showed different antioxidant ability with different reaction mechanisms [15,16]. Therefore, the evaluation of antioxidant activity is a rather difficult task when a single method is selected. For that reason, the antioxidant activity of samples must be evaluated with a variety of methods that can address the different mechanisms.

### 2.2. Total Phenolic, Flavonoid and Proanthocyanidin Contents

Plants have been reported to be rich sources of phytochemicals such as phenolics, flavonoids, and proanthocyanidin, and it has been suggested various health benefits like antioxidant activity [17,18]. In addition, Cho *et al*. [19] reported that various antioxidant activities from natural resources were significantly correlated with their major compound contents, such as polyphenol and flavonoid. Therefore, we determined the total phenolic, flavonoid, and proanthocyanidin contents of extracts obtained from various parts of *A. senticosus* and *A. koreanum*. Table 1 shows the total phenolic content of extracts from different parts of *A. senticosus* and *A. koreanum*. Among the different extracts, the highest phenolic content (73.34 mg GAE/dry weight g) was observed from fruit extracts of *A. koreanum* followed by leaf extracts (56.08 mg GAE/dry weight g), root extracts (44.00 mg GAE/dry weight g) of *A. senticosus* and leaf extracts (40.61 mg GAE/dry weight g) of *A. koreanum*, however, stem extracts of *A. senticosus* exhibited the lowest phenolic contents (23.50 mg GAE/dry weight g). On the other hand, the leaf extracts of *A. senticosus* (41.23 mg RE/dry weight g) and *A. koreanum* (38.21 mg RE/dry weight g) showed higher total flavonoid contents than other part extracts of their plants. In all parts of *A. senticosus*, total phenolic and flavonoid contents followed the same trend; leaf > root > fruit > stem. In another study, the highest amount of total phenolic and flavonoid contents also observed from leaves extracts of *A. senticosus* [20]. In addition, the total phenolic contents of the leaf extracts from *Withania somnifera* (L.) also exhibited as remarkably higher than in the other parts of the plant [21]. Proanthocyanidin content in the fruit extracts of *A. senticosus* (10.86 mg CE/dry weight g) and *A. koreanum* (33.01 mg CE/dry weight g) was higher than other parts (Table 1). Total content of phenolic, flavonoid, and proanthocyanidin influence antioxidant ability [19,22]. Many medicinal plants contain large amounts of antioxidants such as polyphenols [23], and the antioxidant activity of the extracts from various plants might be correlated with their total phenolic compounds [19]. Therefore, the antioxidant activities in different parts of *A. senticosus* and *A. koreanum* were correlated with total phenolic, flavonoid, and proanthocyanidin contents.

**Table 1:** Total phenolic, total flavonoid and proanthocyanidin contents in the different parts of *A. senticosus* and *A. koreanum*.

Sample		Total Phenolic Contents (mg GAE/dry weight g)	Total Flavonoid Contents (mg RE/dry weight g)	Proanthocyanidin (mg CE/dry weight g)
*A. senticosus*	Fruit	25.70 ± 0.69 ^f^	16.44 ± 0.99 ^g^	10.86 ± 0.49 ^c^
	Leaf	56.08 ± 0.47 ^b^	41.23 ± 1.98 ^a^	8.75 ± 1.02 ^d^
	Stem	23.50 ± 0.42 ^g^	11.49 ± 1.49 ^h^	2.88 ± 0.28 ^f^
	Root	44.00 ± 0.14 ^c^	36.49 ± 0.37 ^b,c^	7.61 ± 0.56 ^e^
*A. koreanum*	Fruit	73.34 ± 1.16 ^a^	31.31 ± 0.37 ^d^	33.01 ± 1.02 ^a^
	Leaf	40.61 ± 1.03 ^d^	38.21 ± 0.65 ^b^	6.79 ± 0.28 ^e^
	Stem	35.45 ± 0.47 ^e^	21.18 ± 0.99 ^f^	7.44 ± 0.98 ^e^
	Root	34.40 ± 0.69 ^e^	23.56 ± 1.63 ^e^	3.86 ± 0.28 ^f^

GAE, gallic acid equivalent. RE, rutin equivalent. CE, catechin equivalent. Value are Mean ± SD in triplicate (*n* = 3), means in the same column not sharing a common letter are significantly different (*p* < 0.05) by Duncan’s multiple test. ^abcdefg^ significant differences among various parts of samples.

### 2.3. Relationship between Antioxidant Activity and Antioxidant Compounds

To correlate the results obtained by the different methods, a regression analysis was performed (correlation coefficient = R). In the Table 2, among the various methods used to determine the antioxidant potential, the ABTS radical scavenging activity was significantly correlated with reducing power (R = 0.966), FRAP (R = 0.936) and ORAC (R = 0.911) assays, however, a low correlation was observed between DPPH and FRAP (R = 0.580). The obtained results also significantly correlated to the total phenolic content with reducing power (R = 0.992), ABTS (R = 0.981) and ORAC (R = 0.922) assays, and proanthocyanidin was highly correlated with FRAP (R = 0.896) and ABTS (R = 0.877) assays. On the other hand, the total flavonoid content was correlated with DPPH (R = 0.866), and ABTS (R = 0.539), FRAP (R = 0.321), reducing power (R = 0.717) and ORAC (R = 0.637) assays were uncorrelated with total flavonoid contents. Dudonne *et al*. [24] reported that the total phenolic contents from 30 types of plants significantly correlated with ABTS assay. In general, the antioxidant activity of plant extracts was correlated with their major compounds, such as phenolic compounds, flavonoids, carotenoids, and pigments [25]. These results imply that considerable antioxidant activity except DPPH radical assay from *A. senticosus* and *A. koreanum* may be due to total phenolic and proanthocyanidin contents rather than total flavonoid contents. Therefore, we determined major active compounds of different parts extracts from *A. senticosus* and *A. koreanum* using HPLC analysis.

**Table 2:** Correlation analysis (R) between the antioxidant activity and antioxidant compounds in the different parts of *A. senticosus* and *A. koreanum*.

Parameter	TPC	TFC	PC	DPPH	ABTS	FRAP	Reducing Power	ORAC
TPC	1	0.688	0.802 *	0.867 **	0.981 **	0.876 **	0.992 **	0.922 **
TFC	0.688	1	0.218	0.866 **	0.539	0.321	0.717 *	0.637
PC	0.802 *	0.218	1	0.480	0.877 **	0.896 **	0.812 *	0.769 *
DPPH	0.867 **	0.866 **	0.480	1	0.792 *	0.580	0.861 **	0.759 *
ABTS	0.981 **	0.539	0.877 **	0.792 *	1	0.936 **	0.966 **	0.911 **
FRAP	0.876 **	0.321	0.896 **	0.580	0.936 **	1	0.869 **	0.883 **
Reducing power	0.992 **	0.717 *	0.812 *	0.861 **	0.966 **	0.869 **	1	0.927 **
ORAC	0.922 **	0.637	0.769 *	0.759 *	0.911 **	0.883 **	0.927 **	1

TPC, Total phenolic contents. TFC, Total flavonoid contents. PC, Proanthocyanidin. * The correlation coefficients are significant at the 0.05 level. ** The correlation coefficients are significant at the 0.01 level.

### 2.4. The Chlorogenic Acid and Caffeic Acid Contents

**Figure 2:**
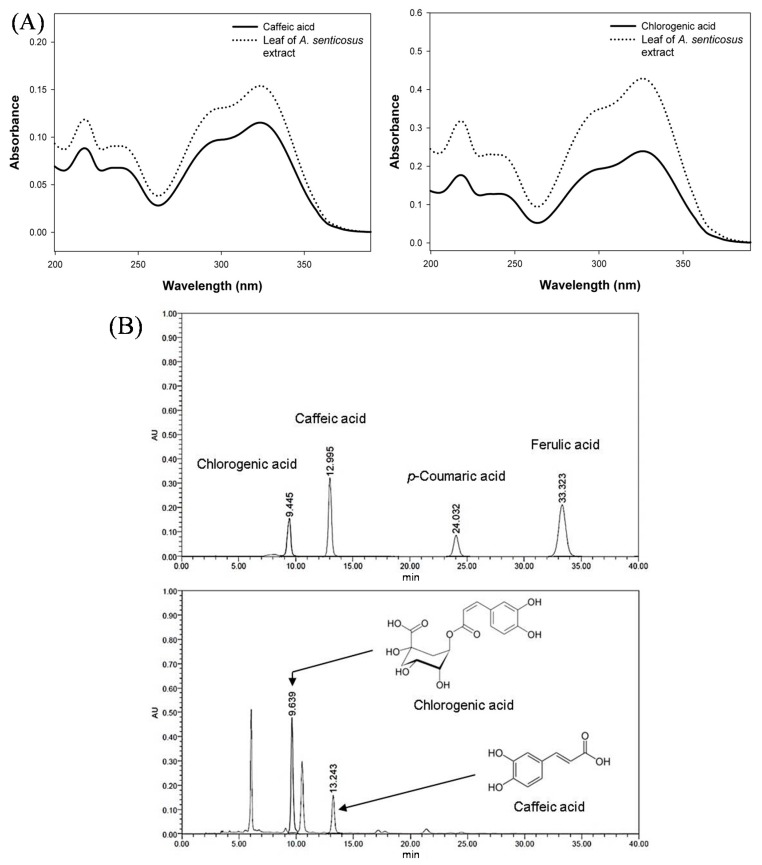
PDA spectrums of caffeic acid and chlorogenic acid (**A**); and HPLC chromatograms of phenolic compounds (chlorogenic acid, caffeic acid, *p*-coumaric acid, and ferulic acid) (**B**) of leaf extract from *A. senticosus.*

The chlorogenic acid and caffeic acid are major compounds in *Acanthopanax* species [6]. The contents of chlorogenic acid and caffeic acid in different parts of *A. senticosus* and *A. koreanum* were analyzed using the developed HPLC method. As a result, chlorogenic acid and caffeic acid were found in extracts from leaf of *A. senticosus*, however, the peak areas corresponding to *p*-coumaric acid and ferulic acid were not found in the sample (Figure 2). Therefore, we determined chlorogenic acid and caffeic acid content in the current study. Table 3 showed chlorogenic acid and caffeic acid contents in different parts of *A. senticosus* and *A. koreanum*. The highest contents of chlorogenic acid and caffeic acid were observed from leaf extracts of *A. senticosus*. Caffeic acid in fruit and stem extracts of *A. senticosus* and fruit extracts of *A. koreanum* was not detected. The chlorogenic acid contents of leaves extracts from *A. senticosus* (14.86 mg/g) and *A. koreanum* (5.43 m/g) were significantly greater than leaves extracts of *A. senticosus* from Mudanjiang (1.1 mg/g) and Wudalianchi (0.23 mg/g) in Heilongjiang province, China [13]. These results suggest that the chlorogenic acid and caffeic acid contents of *Acanthopanax* species may be significantly different in their growing environmental factors such as temperature, light, water, and nutrient availability [26].

The obtained results indicate that the strong antioxidant activities and highest amount of chlorogenic acid and caffeic acid contents from leaf of *A. senticosus* could be a good source for natural antioxidant products. Therefore, the major phenolic compounds were validated by HPLC method from leaf extracts of *A. senticosus*.

**Table 3:** Chlorogenic acid and caffeic acid contents in the different parts of *A. senticosus* and *A. koreanum*.

Sample		Chlorogenic Acid (mg/dry weight g)	Caffeic Acid (mg/dry weight g)
*A. senticosus*	Fruit	0.57 ± 0.01 ^f^	Not detected
	Leaf	14.86 ± 0.12 ^a^	3.09 ± 0.02 ^a^
	Stem	6.02 ± 0.12 ^b^	Not detected
	Root	5.01 ± 0.20 ^d^	0.10 ± 0.03 ^d^
*A. koreanum*	Fruit	1.92 ± 0.03 ^e^	Not detected
	Leaf	5.43 ± 0.16 ^c^	1.61 ± 0.07 ^b^
	Stem	5.96 ± 0.07 ^b^	1.22 ± 0.04 ^c^
	Root	6.14 ± 0.14 ^b^	0.11 ± 0.01 ^d^

Value are Mean ± SD in triplicate (*n* = 3), means in the same column not sharing a common letter are significantly different (*p* < 0.05) by Duncan’s multiple test. ^abcdef^ significant differences among various parts of samples.

### 2.5. Method Validation

#### 2.5.1. Specificity

As recommended by the Korea food and drug administration (KFDA) guidelines [27], a chromatographic method is considered specific (selective) when each peak within the chromatogram corresponds to only one substance. Specificity is the ability to distinguish between the analyte and other substances that may be present in a sample. In general, this analysis is performed by a photo diode array (PDA) detector that provides the chromatogram and ultraviolet (UV) spectral information. As shown in Figure 2, the peak of *A. senticosus* leaf extracts was verified by comparing the retention time in the HPLC chromatogram and the UV spectrum (200–400 nm) of chlorogenic acid and caffeic acid. However, the peak areas corresponding to *p*-coumaric acid and ferulic acid were not found in the leaf extracts from *A. senticosus*.

#### 2.5.2. Evaluation of Accuracy and Precision, Limit of Detection, and Limit of Quantification

The precision of the method was assessed by intra-day and inter-day variations. The method showed good precision, with intra-day and inter-day variations of 0.87%–1.39% (RSD %) and 1.47%–1.70%, respectively (Table 4). To evaluate the accuracy of the method, as shown in Table 5, a recovery study was carried out by spiking *A. senticosus* leaf extracts at three concentrations (25%, 50% and 100% of *A. senticosus* leaf extracts) of chlorogenic acid and caffeic acid. The mean recovery for chlorogenic acid ranged from 105.01%–110.74%, while the percentage of relative standard deviation (RSD) for recovery was 0.92%–3.10%. The mean recovery for caffeic acid ranged from 91.29%–96.41%, while the percentage of RSD for recovery was 0.52%–1.91%. Thus, the developed method was considered to be reproducible and accurate (Table 5). Based on these results, a simple and reliable RP-HPLC method with photodiode assay detector for the quantification of chlorogenic acid and caffeic acid has been developed and validated for its specificity, linearity, accuracy, precision, limits of detection (LOD), and limits of quantification (LOQ).

The calibration curve showed excellent linearity (correlation coefficients (R): chlorogenic acid and caffeic acid = 0.9997 and 0.9991, respectively). The LOD and LOQ values of the chlorogenic acid were 0.10 μg/mL and 0.30 μg/mL, respectively, and the LOD and LOQ values of the caffeic acid were 0.03 μg/mL and 0.06 μg/mL, respectively (Table 5).

**Table 4:** Precision studies of chlorogenic acid and caffeic acid analysis for the determination in the leaf extract of *A. senticosus*.

Parameters	Chlorogenic Acid (mg/dry weight g)		Caffeic Acid (mg/dry weight g)	
	Mean ± SD	RSD (%)	Mean ± SD	RSD (%)
Inter-day	15.15 ± 0.13	0.87	3.32 ± 0.05	1.39
Intra-day	15.56 ± 0.26	1.70	3.43 ± 0.05	1.47

Results are expressed as the mean ± standard deviation (SD) from at least three independent experiments performed in triplicate. RSD, Relative standard deviation. Inter-day, One time analysis of chlorogenic acid and caffeic acid per day for 3 days. Intra-day, Three times analysis per day.

**Table 5:** The recovery, LOD and LOQ values of chlorogenic acid and caffeic acid in the leaf extract from *A. senticosus*.

Added (mg/dry weight g)		Recovery (%)		Values of LOD and LOQ (μg/mL)	
		Mean ± SD	RSD (%)	LOD	LOQ
Chlorogenic acid	15.15 (100%)	110.74 ± 2.84	2.56	-	-
	7.58 (50%)	105.01 ± 0.97	0.92	-	-
	3.79 (25%)	106.00 ± 3.29	3.10	0.1	0.3
Caffeic acid	3.32 (100%)	96.41 ± 1.84	1.91	-	-
	1.66 (50%)	92.49 ± 0.75	0.81	-	-
	0.83 (25%)	91.29 ± 0.47	0.52	0.03	0.06

Results are expressed as the mean ± standard deviation (SD) from at least three independent experiments performed in triplicate. RSD, Relative standard deviation.

## 3. Experimental Section

### 3.1. Sample Preparation

*A. senticosus* and *A. koreanum* were supplied by farms located in Gangwon and Jeju provinces, Korea, respectively. Dried fruit, leaf, stem, and root of *A. senticosus* and *A. koreanum* were ground in a mill and passed through a 35-mesh sieve. Approximately 3 g of dry powdered plant material was extracted with 100 mL water by reflux for 3 h at 100 °C. After filtration using filter paper (Whatman No. 3, Maidstone, Kent, UK), the extract was concentrated by evaporation under a vacuum at 60 °C in a rotary vacuum evaporator (Tokyo Rikakikai Co., Ltd, Tokyo, Japan) and then freeze-dried (Ilshinbiobase Co., Ltd, Gyeonggi, Korea).

### 3.2. Chemicals

The reagents sodium carbonate, aluminum nitrate, potassium ferricyanide, sodium hydroxide, Folin-Ciocalteu’s phenol reagent, vanillin, potassium acetate, Trolox, potassium persulfate, sodium phosphate dibasic, trichloroacetic acid, ferric chloride, phosphoric acid, gallic acid, rutin, catechin, chlorogenic acid, caffeic acid, *p*-coumaric acid, ferulic acid, DPPH (2,2-diphenyl-1-picrylhydrazyl), ABTS [2,2′-azino-bis (3-ethylbenzothiazoline-6-sulfonic acid)], TPTZ (2,4,6-tripyridyl-s-triazine) and AAPH (2,2′-azobis (2-methylpropionamidine) dihydrochloride) were purchased from Sigma Chemical Co. (St Louis, MO, USA). Potassium phosphate monobasic and fluorescein (sodium salt) were purchased from Junsei Chemical Co. (Tokyo, Japan), and HPLC-grade water and acetonitrile were obtained from J. T. Baker (Phillipsburg, NJ, USA).

### 3.3. Antioxidant Activity

#### 3.3.1. DPPH Radical Scavenging Activity

Antioxidant activity determination of the different extracts was performed by the DPPH radical scavenging method [28]. DPPH radicals have an absorption maximum of 517 nm, which disappears with reduction by an antioxidant compound. Water solution (1 mL) containing 1 mg of the freeze-dried extract (0.2 mL) was added to a 0.4 mM DPPH ethanolic solution (0.8 mL). The solution was mixed and allowed to react at room temperature in the dark for 10 min. The absorbance at 517 nm was measured using a microplate reader. The blank was prepared in the same manner, except that distilled water was used instead of the sample. The radical scavenging activity was calculated as a percentage using the following equation:

(1) DPPH radical scavenging activity (%) = [1 − (A_sample_/A_blank_)] × 100

#### 3.3.2. ABTS Radical Scavenging Activity

ABTS radical scavenging activity of the different extracts was measured according to the modified method of Re *et al*. [29]. ABTS stock solution was dissolved in water to a 7.4 mM concentration. The ABTS radical cation (ABTS^+^) was produced by reacting ABTS stock solution with 2.45 mM potassium persulfate and allowing the mixture to stand for 14 h at room temperature in the dark. The ABTS^+^ solution was diluted with ethanol to obtain an absorbance of 0.70 ± 0.02 at 750 nm. After adding 1.0 mL of diluted ABTS^+^ solution (A_750nm_ = 0.70 ± 0.02) to 0.01 mL of sample, the mixture was left at room temperature for 30 min in the dark. The absorbance at 750 nm was measured using a microplate reader. The blank was prepared in the same manner, except distilled water was used instead of the sample. The radical scavenging activity was calculated as a percentage using the following equation:

(2) ABTS radical scavenging activity (%) = [1 − (A_sample_/A_blank_)] × 100

#### 3.3.3. Ferric Reducing Antioxidant Power (FRAP)

The FRAP assay was performed using a modified version of the FRAP assay [30]. The working FRAP reagent was prepared daily by 300 mM acetate buffer (pH 3.6), a 10 mM TPTZ solution in 40 mM HCl and 20 mM FeCl_3_·6H_2_O solution in proportions of 10:1:1 (*v*/*v*), respectively. The FRAP reagent was prepared fresh daily and was warmed to 37 °C in a water bath prior to use. Then, 0.05 mL of sample was mixed with distilled water (0.15 mL) and the FRAP reagent (1.5 mL). The reaction mixture was incubated at 37 °C in a water bath for 4 min, and the absorbance of the colored products was measured at 595 nm.

#### 3.3.4. Reducing Power 

The reducing power of the sample was determined according to the modified method of reducing power assay [31]. This assay is based on reduction; the yellow color of the solution changes to various shades of green and blue depending on the reducing power of sample. The sample (0.1 mL) was added to 0.2 M sodium phosphate buffer (0.5 mL) and 1% potassium ferricyanide (0.5 mL), and this mixture was incubated at 50 °C for 20 min. Following incubation, 10% trichloroacetic acid solution (0.5 mL) was added to the reaction mixture, and it was centrifuged at 12,000 rpm for 10 min. The supernatant was mixed with distilled water (0.5 mL) and a 0.1% iron (III) chloride solution (0.1 mL), and the absorbance at 700 nm of the resulting solution was measured.

#### 3.3.5. Oxygen Radical Absorbance Capacity (ORAC)

The ORAC assay is based on the scavenging of peroxyl radicals generated by AAPH, which prevent the degradation of the fluorescein probe. The ORAC assay was performed using a modified method of Ou *et al*. [32]. The sample was diluted with a 75 mM potassium sodium phosphate buffer (pH 7.4), and 0.025 mL of either the diluted Trolox (0–10 μM) or the diluted sample, together with 150 μL of 40 nM fluorescein, was added into black-walled 96-well plate. Finally, 25 μL of 18 mM AAPH was pre-incubated at 37 °C for 15 min and transferred to each well, and the plate was immediately carried to the fluorescence microplate reader (Spectramax GEMINI EM, Molecular Devices, Sunnyvale, CA, USA) to measure the fluorescence. The analyzer was set to an excitation wavelength of 485 nm and an emission wavelength of 530 nm, and readings were recorded every 3 min for 90 min at 37 °C. The ORAC value results were calculated using a Trolox calibration curve and the area under the fluorescence decay curve. The ORAC value was expressed as Trolox equivalents in micromoles per milliliter.

(3) Area under the curve (AUC) = 1 + f1/f0 + f2/f0 + f3/f0 +f4/f0 + ...f31/f0

#### 3.3.6. Total Phenolic Content (TPC)

The total phenolic content was determined using Folin-Ciocalteu assay [33]. Water solution (1 mL) containing 1 mg of the freeze-dried extract or standard was mixed with 1 mL of 2% sodium carbonate solution and 1 mL of 10% Folin-Ciocalteu’s phenol reagent. After 90 min, the absorbance was measured at 750 nm using a microplate reader (Molecular Devices, Sunnyvale, CA, USA). The measurement was compared to calibration curve of gallic acid [total phenolic concentration = (absorbance − 0.0136)/15.177 × extraction yield; R^2^ = 0.996; *p* < 0.001], and the results were expressed as milligrams of gallic acid equivalents (GAE) per gram of sample [mg GAE/g].

#### 3.3.7. Total Flavonoid Content (TFC)

The total flavonoid content was determined using a method described by Zhishen *et al*. [34] with modifications. Water solution (0.5 mL) containing 0.5 mg of the freeze-dried extract was mixed with 1.5 mL of ethanol, 0.1 mL of 10% aluminum nitrite solution, 0.1 mL of 1 M potassium acetate solution and 2.8 mL distilled water. The mixture was stirred and allowed to react for 30 min. The absorbance was then measured at 415 nm using a microplate reader. The measurement was compared to a calibration curve of rutin [flavonoid concentration = (absorbance + 0.0001)/1.5467 × extraction yield; R^2^ = 0.999; *p* < 0.001], and the results were expressed as milligrams of rutin equivalents (RE) per gram of sample [mg RE/g].

#### 3.3.8. Proanthocyanidin Content (PC)

The proanthocyanidin content was determined using vanillin-hydrochloric acid (V-HCl) method [35]. Methanol solution (0.5 mL) containing 0.5 mg of freeze-dried extract was placed in a brown tube, and 3 mL of 4% vanillin solution was added. After stirring vigorously, 1.5 mL of concentrated hydrochloric acid was added to the mixture and was allowed to react for 15 min. The absorbance was then measured at 490 nm using a microplate reader. The measurement was compared to a calibration curve of catechin [proanthocyanidin concentration = (absorbance − 0.0011)/2.0467 × extraction yield; R^2^ = 0.999; *p* < 0.001], and the results were expressed as milligrams of catechin equivalents (CE) per gram of sample [mg CE/g].

### 3.4. HPLC Analysis of Phenolic Compounds

Analysis of the phenolic compounds was carried out with an HPLC system (Waters 2695 Separation Module, Waters Co., Milford, MA, USA) controlled by Empower software. Polyphenol separation was determined using a modified method of Li *et al.* [12].

For the simultaneous analysis of chlorogenic acid, caffeic acid, *p*-coumaric acid and ferulic acid were identified by comparing the retention times and UV spectra with those obtained with standard mixture. The phenolic compounds were quantified by the external standard method with calibration curves (correlation coefficients > 0.999 for chlorogenic acid and caffeic acid).

### 3.5. Validation Procedure

The HPLC method was validated in terms of linearity, specificity, limit of detection (LOD), limit of quantification (LOQ), precision, and accuracy according to the guidelines of KFDA (Korea Food & Drug Administration) [27].

#### 3.5.1. Specificity

The specificity of the method was assessed by comparing the chromatograms (HPLC) and PDA spectra (UV) obtained from the standard phenolic compounds and the leaf extract from *A. senticosus*.

#### 3.5.2. Linearity

For the linearity study, calibration curves were carried out with five concentration levels for each target. Each concentration was analyzed three times. The calibration curve was quantified as y = Ax + B, where “A” is the slope of the calibration curve, “B” is the intercept of the calibration curve, “x” is the concentration of the phenolic compound in standard solution, and “y” is the peak area. The correlation (R) value indicated linearity.

#### 3.5.3. Precision and Accuracy

Precision was evaluated by measuring analysis repeatability in the intra- and inter-day tests. The samples were injected three times, and the results were expressed as the Relative Standard Deviation of measurements (RSD %).

Accuracy was validated through a spike recovery test. To perform this test, sample solutions were prepared by adding phenolic compound at three different concentration levels (25%, 50%, 100% of phenolic compound from *A. senticosus* leaf extract). The accuracy was expressed as the percentage of the amount recovered in the spiked compared to the known concentration.

#### 3.5.4. Limit of Detection (LOD) and Limit of Quantification (LOQ)

The LOD is defined as the lowest concentration of an analyte that an analytical process can reliably differentiate from background levels. Additionally, the lowest concentration that can be quantified with acceptable accuracy and precision is defined as LOQ. The LOD and LOQ of the HPLC method were estimated from the signal-to-noise ratio (S/N). The LOD and LOQ for each analyte were estimated as the concentration levels at which the S/N reached 3 and 10, respectively.

### 3.6. Statistical Analysis

All data are presented as means ± SD. The data were analyzed using the one-way ANOVA procedure of SAS 9.3 (SAS Institute Inc., Cary, NC, USA). Differences were analyzed using Duncan’s multiple range tests at a *p-*value < 0.05 level. Correlations were calculated using Pearson’s correlation coefficient (R) by the IBM SPSS Statistics software package (IBM SPSS Statistics 21, IMB, NY, USA).

## 4. Conclusions

In this study, the antioxidant activities of different parts extracts from *A. senticosus* and *A. koreanum* were measured, and total phenolic, flavonoid, and proanthocyanidin contents are determined. In addition, the major phenolic compounds (chlorogenic acid and caffeic acid) were validated using HPLC method. The *in vitro* antioxidant activities observed that fruit extracts from *A. senticosus* and *A. koreanum* showed highest ABTS, FRAP, reducing power and ORAC capacity, however, leaf extract of *A. senticosus* exhibited strongest DPPH radical scavenging activity. The antioxidant activities from various extracts correlated with total phenolic and proanthocyanidin contents rather than total flavonoid contents. The major phenolic compounds from *A. senticosus* and *A. koreanum* were identified as chlorogenic acid and caffeic acid by HPLC. Moreover, the leaf of *A. senticosus* higher contents of chlorogenic acid and caffeic acid than other part of their plants. Therefore, the leaf of *A. senticosus* is an excellent natural source for antioxidants and can be utilized to develop functional foods as well as serve health-promoting pharmaceutical agents, and the developed validation method was useful for the quality control of *Acanthopanax* species.
